# Measurement Comparability of Electronic and Paper Administration of Visual Analogue Scales: A Review of Published Studies

**DOI:** 10.1007/s43441-022-00376-2

**Published:** 2022-02-10

**Authors:** Bill Byrom, Celeste A. Elash, Sonya Eremenco, Serge Bodart, Willie Muehlhausen, Jill V. Platko, Chris Watson, Cindy Howry

**Affiliations:** 1 Signant Health, Ground Floor, Waterfront Embankment, Manbre Road, Hammersmith, London, W6 9RH UK; 2 YPrime, Malvern, PA USA; 3 Critical Path Institute, Tucson, AZ USA; 4 IDDI, Ottignies, Belgium; 5 Safira Clinical Research Ltd, Nenagh, Ireland; 6 Signant Health, Blue Bell, PA USA; 7 THREAD, Cary, NC USA; 8 assisTek, Doylestown, PA USA

**Keywords:** Electronic patient-reported outcome, ePRO, Measurement comparability, Measurement equivalence, Visual analogue scale, VAS

## Abstract

**Background:**

Visual analogue scales (VASs) are used in a variety of patient-, observer- and clinician-reported outcome measures. While typically included in measures originally developed for pen-and-paper completion, a greater number of clinical trials currently use electronic approaches to their collection. This leads researchers to question whether the measurement properties of the scale have been conserved during the migration to an electronic format, particularly because electronic formats often use a different scale length than the 100 mm paper standard.

**Methods:**

We performed a review of published studies investigating the measurement comparability of paper and electronic formats of the VAS.

**Results:**

Our literature search yielded 26 studies published between 1997 and 2018 that reported comparison of paper and electronic formats using the VAS. After excluding 2 publications, 23 of the remaining 24 studies included in this review reported electronic formats of the VAS (eVAS) and paper formats (pVAS) to be equivalent. A further study concluded that eVAS and pVAS were both acceptable but should not be interchanged. eVAS length varied from 21 to 200 mm, indicating that 100 mm length is not a requirement.

**Conclusions:**

The literature supports the hypothesis that eVAS and pVAS provide comparable results regardless of the VAS length. When implementing a VAS on a screen-based electronic mode, we recommend following industry best practices for faithful migration to minimise the likelihood of non-comparability with pVAS.

## Introduction

**Fig. 1:**
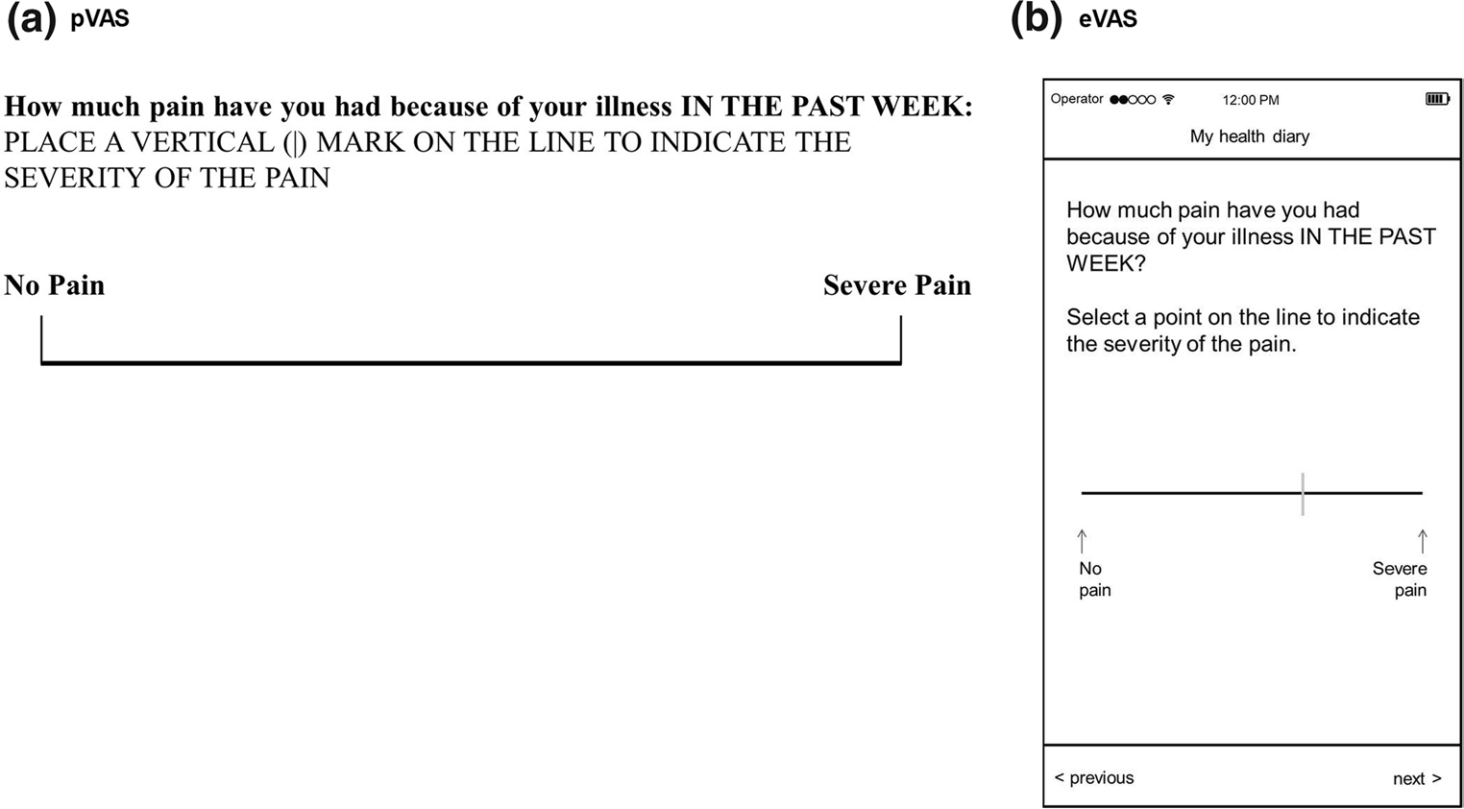
Typical representations of pVAS and eVAS based on The Stanford Health Assessment Questionnaire Disability Index HAQ-DI [46]

Visual analogue scales (VASs) are commonly used in the assessment of a variety of health-related constructs including pain [1–3], mood [4], quality of life [5, 6], and patient satisfaction [7], and have been found to provide reliable and valid data [8]. The VAS is defined in the FDA’s Guidance for Industry on Patient-Reported Outcome Measures [9] as “a line of fixed length (usually 100 mm) with words that anchor the scale at the extreme ends and no words describing intermediate positions” (Fig. 1). VASs are brief and simple to complete and are particularly useful when assessing a single construct with many perceptible gradations due to the continuous nature of the scale and the 101 possible response options. These characteristics also contribute to the scale’s high sensitivity to change [10].

### VAS Acceptability and Utility

Because the VAS uses few words, the vocabulary level of the respondent is generally not a concern, provided the anchor descriptors are straightforward, common terms [11]. However, some research has demonstrated poor reliability of the VAS in illiterate patients [12].

People with visual impairments can typically see a VAS easily. Most respondents have sufficient dexterity to use a VAS [11], although those with dexterity issues, for example due to arthritis or orthopaedic trauma to their hands or arms, may have difficulty targeting a very specific point on the scale to indicate their desired response [10].

Some research indicates children younger than 6 or 7 may have difficulty responding to a VAS [13]. In addition, older adults may have more difficulty responding to a VAS than younger people [10], with some research showing increased age associated with incorrect response on a paper VAS [14].

The VAS has additional limitations related to the visual nature of the scale. For example, a VAS is not appropriate for telephone interview-based or interactive voice response (IVR) system- or voice assistant-based data collection because the respondent must be able to see the scale in order to select a response.

### Implementation Formats

Traditionally, the VAS has been implemented on paper (pVAS) with a 100 mm (10 cm) line to facilitate measurement of 101 discrete points (0 to 100) with a metric ruler [15]. This implementation can introduce sources of error. Measurement of the score on paper with a metric ruler may introduce human error [11] and additional scoring error may be introduced if the response is ambiguous, i.e. not a clear mark within the confines of the scale anchors [8]. Additionally, photocopying the pVAS can change the length of the line, making the comparison between distances measured on the original and the photocopied version difficult [10, 14].

These limitations of the pVAS can be mitigated by implementation on a screen-based electronic platform (Fig. 1). An electronic VAS (eVAS) can be implemented on a line of any length, provided the response fields allow for selection of precisely 101 discrete points comprising an equal number of pixels on the line. With an eVAS, the pixels along the VAS line are invisibly categorised into 101 equal regions so that the position of the response can be converted into a numeric score from 0 to 100. This approach prevents ambiguous or invalid responses and eliminates the need for manual measurement with a ruler, hence removing that source of human error.

### Migrating from Paper to Electronic Formats

Because many existing patient-reported outcome measures (PROMs) were developed and validated on paper, care is needed when migrating them to electronic formats to ensure that the original measurement properties of the measure are unchanged, and that the electronic format is usable in the target group of respondents [16]. Best practice for VAS migration is to present it as a horizontal line with both the item stem and its response scale visible on a single screen, including anchor text (Fig. 1b) and typically with no numbers presented at the anchor positions [17, 18]. If the screen size permits, anchor text should be located before and after the measurement line. If screen space does not allow this, the anchor text can appear above or below the horizontal line but should include an indicator (such as a short vertical line [Fig. 1a] or arrow [Fig. 1b]) to inform the respondent to which location the text corresponds. [17].

To increase the available screen size, it may be possible to display the screen in landscape orientation, although users find automated switching in screen orientation within an assessment inconvenient [18] and some instrument owners may request additional testing (cognitive interview/usability assessment) if a change in screen orientation is introduced. The proximity to the edge of the screen of the start and end of the VAS line should enable easy selection with a finger or stylus.

In some representations, the scale appears with short vertical bars at the ends, indicating the ends of the scale. In general, a VAS presented on a mobile device will be shorter than the typical 10 cm pVAS, but as noted, the eVAS should span sufficient pixels to return a numeric value between 0 and 100 with all integer values possible. eVAS line width and marker thickness should be sufficient for clear visibility and fine position selection.

Many variants of the standard VAS (as described above) exist, including the incorporation of numbers at the anchor points, a numeric display to indicate the value associated with the point on the scale selected (electronic implementations), additional scale gradations, anchor descriptors associated with points on the scale other than the endpoints, and vertical orientation. The use of numbers at the anchor points of an eVAS is not recommended unless the original pVAS also displays a number along with anchor text at each end of the scale. Presentation of a numeric value associated with the point on the line selected is not recommended when migrating a VAS from paper to electronic format. The addition of a numeric indicator changes the measurement properties of the scale from a purely visual representation to a combination of a VAS and a 101-point numeric rating scale. The properties of such changes are unknown.

Although the eVAS is now commonplace, despite current published evidence of comparability between PROM administration formats that include VAS amongst other response scale types [19–21], regulators and researchers continue to question the comparability of eVAS and pVAS specifically, and ePRO versions with shorter lengths than the 10 cm pVAS leading to the need for a focussed publication on that response type specifically.

The purpose of this review was to assess whether there is sufficient evidence in the published literature to claim that eVASs are comparable to pVASs and to determine whether eVAS implementation and its length might affect PROM measurement properties.

## Methods

We performed a literature search for studies evaluating the eVAS compared to the pVAS. The search was conducted using the OneSearch library database provided by Nottingham Trent University (Nottingham, UK) [22], and used the following search terms: “visual analogue scale” and “equivalence” or “validation.” This list of articles was supplemented by additional published equivalence studies in which eVAS comparisons could be isolated that were referenced in published meta-analyses investigating measurement equivalence of electronic and paper PROMs [20, 21] or elsewhere.

Publications identified by the search were inspected. Articles were excluded from the review if they omitted reporting conclusions or data in relation to the comparability of pVAS and eVAS formats, or if the eVAS implementation was seen to significantly depart from best practice recommendations defined by the Critical Path Institute’s ePRO Consortium [17].

The following information (when available) was catalogued from each article: participant characteristics including disease indication and age range, sample size, eVAS and pVAS length, and PROMs studied. In one study [23], the length of eVAS was estimated from the photograph of the device displaying the eVAS presented in the publication and the known dimensions of the device. Qualitative author conclusions from each evaluation were synthesised and, where reported, quantitative measures of equivalence between the pVAS and the eVAS were recorded and summarised.

## Results

Our literature search yielded a total of 26 studies. These were published between 1997 and 2018 and reported comparisons of paper and electronic formats using the VAS to measure aspects of health. Two studies were subsequently excluded based on the previously defined criteria. The first provided usability assessment of the two formats but did not assess measurement equivalence [24]. The second implemented the eVAS in an unconventional way (the marker was a large ball that could be positioned along a wide horizontal strip) [25] and was excluded as this departed considerably from best practice guidelines for electronic implementation [17]. While the authors of this study, exploring the use of an eVAS to measure pain, concluded there were no clinically relevant differences between the measures recorded on all formats studied (paper, laptop, and smartphone), they did report statistically significant differences in mean pain scores between the smartphone implementation and both paper and tablet VAS implementations (mean difference between eVAS (smartphone) and pVAS: 1.93 mm ± 0.46 mm). This bias may be a direct result of the VAS presentation used which may potentially be associated with more difficulties in accurate score positioning when used on the smaller smartphone display format. This underlines the importance of implementing the eVAS using ePRO design best practice as described above.

The 24 studies included in our evaluation [1–4, 23, 26–44] are summarised in Table 1.

**Table 1:** Summary of articles reviewed

Refs.	n	Population	Age	pVAS	eVAS	Electronic mode	PROM	Association measured
[1]	30	Rheumatoid arthritis	Range: 49–70	100 mm	NR	PDA	Pain, fatigue, global health	*r* = 0.86–0.93
[2]	38	Rheumatoid arthritis	Mean: 58 [SD = 13]	100 mm	NR	PDA	Pain, fatigue, global health	NR
[3]	155	Chronic pain	Range: 19–69	100 mm	40–80 mm	SP, Tablet	Pain	ICC = 0.94
[4]	71	Panic disorder and healthy	Range: 17–72	100 mm	200 mm	PC	Anxiety	*ρ* = 0.98
[23]	200	Chronic pain	Mean: 56.5 [SD = 14]	100 mm	50 mm (EST)	PDA	Pain	NR
[44]	28	Haemodialysis	Mean: 61 [SD = 17]	100 mm	100 mm	PC	Appetite	*r* = 0.572–0.770
[26]	35	General population	Range: 22–62	100 mm	40 mm	FP	NR	NR
[35]	12	General population	Range: 8–10	100 mm	24 mm	SW	Appetite	*r* = 0.65–0.75
[36]	12	General population	Mean: 30 [SD = 12]	100 mm	66 mm	PDA	Appetite	NR
[37]	65	General population	Range: 19–54	100 mm	21 mm	FP	Alcohol effects	ICC = 0.96
[27]	30	General population	Medians: 34 (M), 31 (F)	100 mm	70 mm	PDA	Appetite	NR
[28]	22	General population	Range: 56–86	100 mm	100 mm	Tablet	Pain	*R*^2^ = 0.9998
[29]	24	General population	Range 19–57	100 mm	50 mm	PDA	NR	*R*^2^ = 0.997–0.999
[30]	88	Rheumatoid arthritis, ankylosing spondylitis and psoriatic arthritis	Mean: 54 [SD = 11]	NR	NR	PC	Pain	ICC = 0.867–0.943
[31]	189	Chronic pain	Range: 18–82	NR	NR	PC	SF-MPQ (pain intensity)	*ρ* = 0.68
[32]	86	Non-small cell lung cancer	NR	100 mm	53 mm	PDA	LCSS	ICC = 0.645–0.893 *r* = 0.651–0.847
[33]	355	NR	NR	NR	50–800 pixels	PC	NR	NR
[34]	104	Multiple sclerosis and general population	Mean: 49 [SD = 9]	100 mm	100, 200 mm	SP, Tablet	NR	NR
[38]	20	General population	Means: M: 37 [SD = 13] F: 32 [SD = 9]	100 mm	52 mm	PDA	Appetite	*R*^2^ = 0.671–0.868
[39]	30	Osteoarthritis (knee)	Range: 46–77	100 mm	NR	PC	WOMAC v3	ICC: 0.87–0.95
[40]	59	Rheumatoid arthritis (RA)	Range (RA): 26–86	100 mm	200 mm	PC	RA: Pain, fatigue, global health	RA: ICC: 0.955–0.972 *ρ* = 0.963–0.973
	52	Ankylosing spondylitis (AS)	Range (AS): 21–86				AS: pain, fatigue, BASDAI	AS: ICC: 0.922–0.960 *ρ* = 0.919–0.970
[41]	43	Rheumatoid arthritis	Range: 32–83	100 mm	40 mm	PDA	SF-MPQ, pain, fatigue, global health, HAQ-DI	ICC: 0.77–0.93
[42]	45	Rheumatoid arthritis	Range: 25–83	NR	NR	PC	Pain, fatigue, global health	ICC: 0.63–0.857
[43]	43	Rheumatoid arthritis	Range: 18–75 +	100 mm	NR	PC	Pain, fatigue, global health	ICC: 0.833–0.941

*NR* not reported, *EST* estimated length from image of device in publication, *FP* feature phone, *PROM* patient-reported outcome measure, *PC* personal computer, *PDA* = personal digital assistant, *SP* smartphone, *SW* SmartWatch, *SF-MPQ* Short form McGill Pain Questionnaire, *LCSS* Lung Cancer Symptom Scale, *WOMAC* Western Ontario and McMaster Universities Osteoarthritis Index, *BASDAI* Bath Ankylosing Spondylitis Disease Activity Index, *HAQ-DI* Health Assessment Questionnaire Disability Index

### Populations Studied

Eight studies (33%) were conducted in general population volunteers; the remainder included people diagnosed with rheumatoid arthritis (5/24, 21%), chronic pain (3/24, 12.5%), and single studies in people diagnosed with panic disorder, osteoarthritis of the knee, haemodialysis, and non-small cell lung cancer. Three further studies examined multiple populations: rheumatoid arthritis and psoriatic arthritis [30]; multiple sclerosis and general population volunteers [34]; and rheumatoid arthritis and ankylosing spondylitis [40]. One study did not report the participant population studied [33].

One study was conducted in 8- to 10-year-olds [35], and the remaining studies included adults up to 86 years old. All studies were crossover comparisons of paper to at least one form of electronic data collection. Studies varied in size from 12 to 355 participants (median: 43 participants).

### Constructs Measured

VAS items included measures of pain, fatigue, global health, appetite, anxiety, osteoarthritis symptoms, activities of daily living, and alcohol effects. Most studies examined more than one construct, and therefore reported multiple VAS comparisons.

### Electronic Implementations Studied

**Fig. 2:**
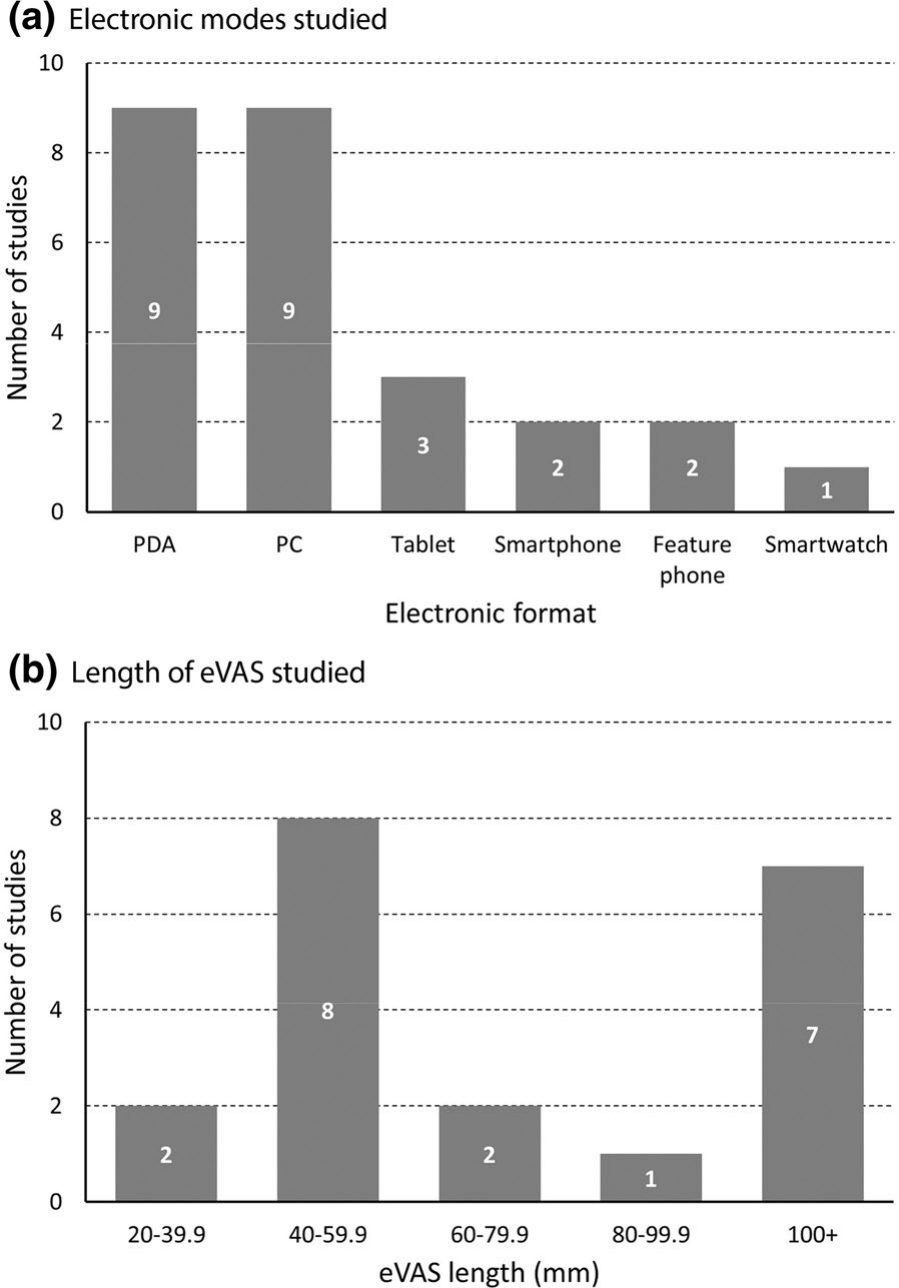
Summary of eVAS implementations. **a** Two studies included two different electronic formats. **b** Seven studies did not report (or enable estimation of) eVAS length

Electronic formats included: personal digital assistant (PDA) (*n* = 9), personal computer (PC) (*n* = 9), tablet (*n* = 3), smartphone (SP) (*n* = 2), feature phone (FP) (a phone with a small, simple display without touchscreen capabilities, *n* = 2), and smartwatch (SW) (*n* = 1) (Fig. 2a), with 2 studies including two electronic formats in addition to paper. One study described the hardware used to display the eVAS as a handheld computer, which we assumed to be a PC due to the year of the article and length of the eVAS [44]. eVAS length was not reported for 7 studies, and where reported ranged from 21 mm on a feature phone screen [37] to 200 mm on a PC implementation [4, 40].

The most common eVAS lengths studied ranged from 40 to 59.9 mm (*n* = 8, Fig. 2b), corresponding with typical smartphone and PDA formats.

### Author Conclusions

Authors of 23 of the 24 studies (96%) concluded that the eVAS and pVAS administrations were comparable. One study (4%), assessing appetite amongst haemodialysis patients using three VAS scales to assess hunger, desire to eat, and fullness [44], concluded that both pVAS and eVAS administrations were suitable to use but were not equivalent and should not be used interchangeably. In this study, the correlations between paper and electronic formats were moderate to strong (0.572, 0.649, and 0.770, Table 1) but in all cases there was a bias towards lower scores on the eVAS. While it is not possible to examine whether the implementation of this study followed electronic patient-reported outcome (ePRO) design best practices, the authors reported that 25% of participants indicated they would not be happy to use the electronic system again, with one usability issue being the quality of visual text displayed.

Two studies (8%) reported trends towards higher scores on eVAS vs. pVAS [1, 2], and five studies (21%) reported the opposite finding towards lower scores on eVAS [4, 28, 35, 39, 44]. Two studies (8%, Apple Newton and Palm PDA device) indicated eVAS scores may be lower than pVAS scores at the scale ends [36, 38]. A later study (using a Dell Pocket PC PDA device) reported no scale-end effects [27].

Authors of all articles concluded that differences between eVAS and pVAS were not clinically relevant, although in five articles (21%) they suggested that paper and electronic forms should not be used interchangeably [35, 36, 38, 39, 44].

### Quantitative Evaluations

**Fig. 3:**
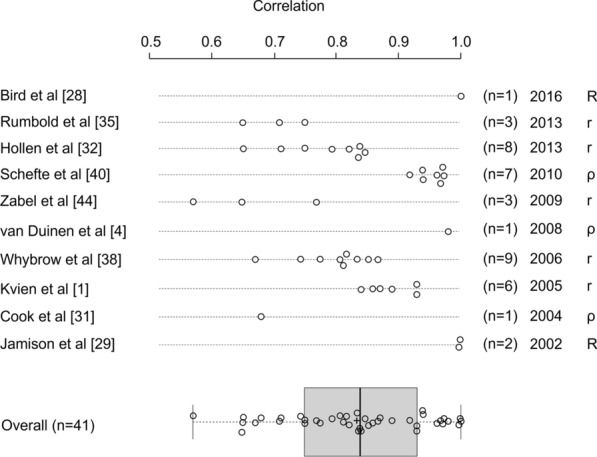
Summary of correlation analyses comparing eVAS and pVAS scores (*n* = 41). *r *Pearson’s *r*, *R*^2^ coefficient of determination, ρ spearman’s rho. *R*^*2*^ values presented as *R* values in this figure. Box plot description: box = interquartile range; box divider = median; “+” = mean; start and end of whisker lines: minimum and maximum values, respectively

Seventeen studies (71%) reported a measure of statistical association or correlation between eVAS and pVAS scores, with the remaining seven studies not reporting measures of association. Of the 10 studies reporting a measure of correlation, Pearson’s *r* (*r*), coefficient of determination (*R*^2^) or Spearman’s rho (*ρ*) values ranged from 0.57 to 0.99, with 85.4% of correlations exceeding 0.7. Authors reported moderate correlations (0.5 to 0.7) in at least 1 VAS comparison in 5/10 studies (50%), representing 6/41 (14.6%) reported correlations; and authors reported strong correlations (> 0.7) in some or all comparisons in 9/10 studies (90%), representing 35/41 (85.4%) reported correlations (Table 1; Fig. 3).

**Fig. 4:**
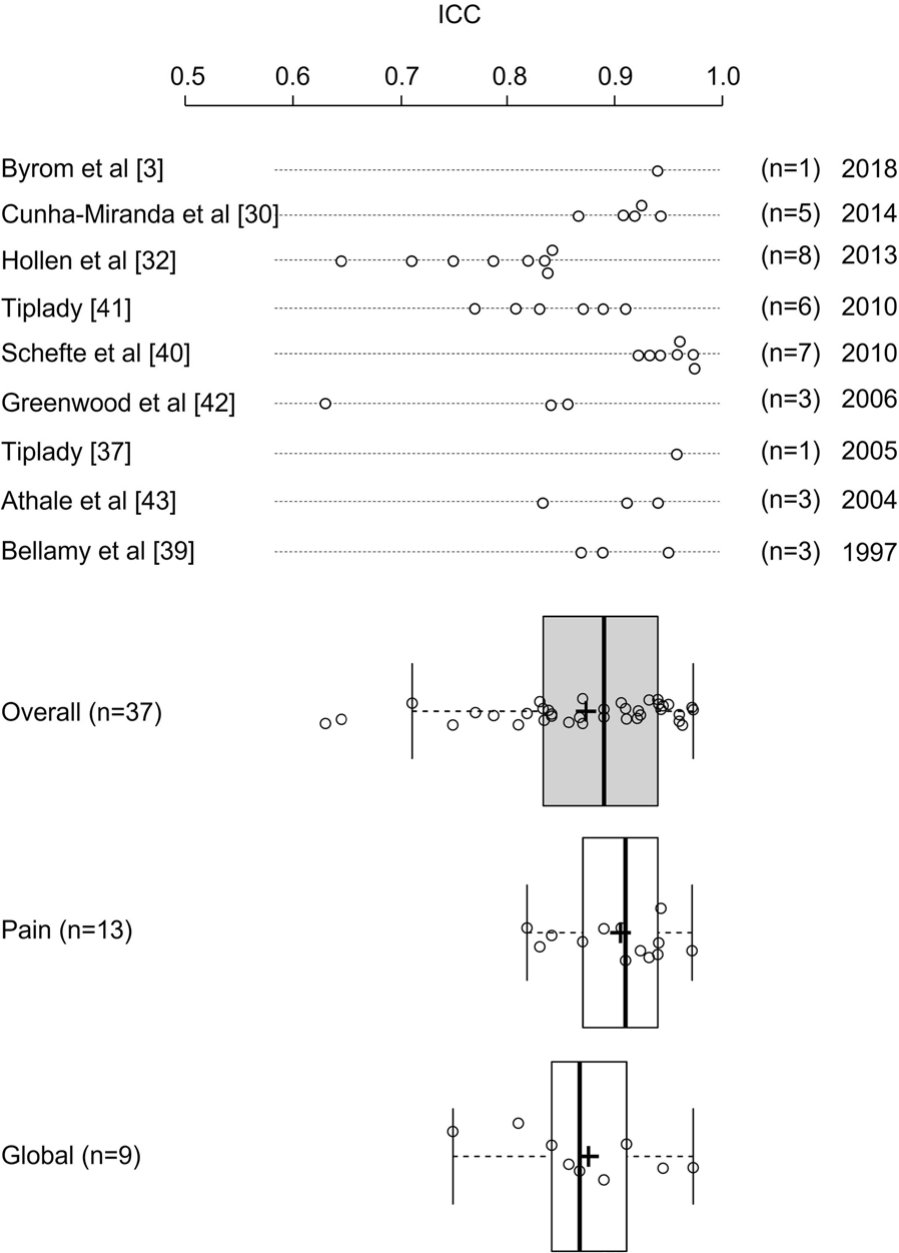
Summary of ICC analyses comparing eVAS and pVAS scores (*n* = 37). Box plot description: box = interquartile range; box divide* r* = median; “+” = mean; start and end of whisker lines: minimum and maximum values, respectively

More appropriate measures of association between pVAS and eVAS include the intraclass correlation coefficient (ICC), reported by nine studies (including two studies reporting both *r*/*ρ* values and ICCs). ICC values exceeded 0.7 for all comparisons in 7/9 (77.8%) studies and for 35/37 (94.6%) individual comparisons reported. In one study examining the equivalence of PDA and paper VAS administration using nine VAS items in people living with non-small cell lung cancer, one VAS item assessing haemoptysis achieved an ICC of 0.645, with the other eight VAS items associated with ICC values above 0.7 (0.711 to 0.893) [32]. A second study in people living with rheumatoid arthritis reported ICCs for paper vs electronic administration of 0.63, 0.841,and 0.857 for fatigue, pain, and patient global assessment, respectively [42]. Across the 37 reported ICC values, the overall mean [SD] ICC was 0.869 [SD = 0.086], with lower and upper quartiles of 0.833 to 0.940, respectively (Fig. 4).

Considering the ICCs comparing pVAS to eVAS for measures of pain (*n* = 13) and patient global assessment (*n* = 9) separately, means [SD] of reported values were 0.901 [SD = 0.048] and 0.871 [SD = 0.069], respectively, with distributions represented in Fig. 4. Reported correlations between pVAS and eVAS appeared consistent independent of the age of publication (Figs. 3 and 4), suggesting that improvements in technology and its familiarity do not appear to impact the measurement comparability between formats.

## Discussion

No clinically relevant difference between pVAS and eVAS was reported by authors of studies identified for inclusion within this literature review. Some scale-end effects were reported in the studies using an Apple Newton and a Palm Pilot system for data collection. These are relatively old technologies, and the design of the device may have led to this effect. While some variability in association measures was observed, the consolidated evidence is broadly supportive that pVAS and eVAS are associated with comparable measurement properties.

Current ePRO implementations are typically performed using smartphone and tablet devices. While our review included only 3 studies using these device types [3, 28, 34], these were amongst the strongest associations reported. Of the two reporting quantitative comparisons, Byrom et al. [3] reported an ICC of 0.94 (95% confidence interval: 0.93–0.96) which included comparison of pVAS to eVAS on both provisioned smartphone and tablet devices and bring-your-own-device with varying device sizes and screen resolutions; and Bird et al. [28] reported an *R*^2^ value of 0.9998.

In addition, based on the publications reviewed, the length of the eVAS does not appear to affect its measurement properties, even for very short VAS lengths.

Encouragingly, the ICC values reported in our evaluation are broadly in line with other evaluations of comparability of PROM formats. This provides some face validity to the sample of studies included. The range of ICCs we report (0.63–0.96) is broadly comparable with the ranges of correlations reported by Muehlhausen et al. [20] and Gwaltney et al. [21] in their meta-analyses of paper and electronic PROMs (ranges: 0.65–0.99 and 0.67–0.98, respectively), although these formal meta-analyses did not distinguish individual response scale types.

Researchers should note, however, that best practices for ePRO design should be implemented when applying the VAS on an electronic medium. These are well described in the output of the ePRO Consortium [17], Oxford University Innovation [45], and industry textbooks [18] and summarised for completeness in Table 2.

**Table 2:** Best practice for eVAS display implementation [17]

Item	Description
Orientation	Present in a horizontal not vertical format
	Avoid switching between portrait and landscape orientations within a measure
Single item per page	Display the full item stem and response scale together on a single screen
Anchor text	If screen size is sufficient, present anchor text (verbal descriptions of the meaning of the ends of the scales) horizontally before and after the measurement line. Where this is not optimal based on screen size, place anchor text under the start and end of the VAS line, and use sentence wrapping and consider arrows to make it clear that the anchors refer to the very start and end of the scale
Measurement attributes	The scale marker should be fine enough to enable precise location, but distinct enough to be clearly visible
	Enable both tapping and sliding to select/adjust the marker location on the line
	Ensure sufficient space between the edge of the screen display and the start/end of the VAS to make it possible to select the very ends of the scale using a finger tap
	The eVAS should return an integer value from 0 to 100. The scale must be composed of a sufficient number of pixels to enable any integer value in the 0 to 100 range to be selected. Each integer value should be associated with the same number of pixels on the measurement line
	The eVAS should not present numbers at the ends of the scale, or visually report the numeric rating associated with the marked point on the scale to the respondent

Recent review articles [8, 15] suggest superiority of the numeric rating scale (NRS) over the VAS in adult pain measures because it was found easier to administer and score, and had both higher respondent acceptability and better psychometric properties. Electronic solutions mitigate the administration and scoring challenges associated with paper implementation of the VAS, but respondent acceptability and psychometric properties remain important considerations when considering the use of the VAS. These review articles also suggest the VAS to be more difficult for certain respondent populations to understand. While we believe this is likely, more evidence is needed to substantiate this assertion. However, while electronic tools simplify the implementation of the VAS, concerns over comprehension is one reason why new instrument developers typically select other measurement scales as opposed to the VAS.

### Limitations of this Research

This literature review was not intended to be a comprehensive review of evidence regarding the comparison of eVAS with pVAS. Rather, it was designed to address whether there is sufficient evidence available to claim that pVASs are comparable to eVASs and to assess whether or not the length of the eVAS influences its measurement properties. While a modest number of studies (*n* = 17) provided a quantitative comparison of pVAS and eVAS, a formal meta-analysis was not possible due to the limitations in statistical reporting in the majority of the published studies included. For example, only 1 of the 9 studies reporting ICCs also reported ICC confidence intervals. [3]

Seven articles (29%) did not report eVAS length. Despite this, the remaining articles reported comparisons of the 100 mm pVAS to eVAS lengths ranging from 21 to 200 mm. It would be helpful to ensure that VAS lengths are reported in future studies comparing the measurement properties of eVAS and pVAS scales.

Twelve articles (50%) were at least 10 years old and only 5 articles (21%) were published in the last 5 years. Although screen resolutions have improved substantially, there is no reason to believe that the eVAS measurements from older devices are not comparable to those collected using more modern devices. However, as discussed above, the recessing of the screen within the hardware casing of the Apple Newton and Palm PDA devices may have contributed to the scale-end effects reported using those devices. In addition, only 5 studies investigated electronic administration of the VAS on a smartphone, a tablet, or a smartwatch. Considering the recent improvements in electronic data collection methods and technologies for clinical trials, it would be helpful to review more recent literature to corroborate these findings with current technology.

This research is general and does not focus on specific therapeutic areas or populations. While one study presented findings in children aged 8 to 10 years, we found few published studies including the use of VAS by older adults, children, or people with visual impairment or other physical restrictions.

Authors did not provide detailed descriptions of the display properties of the measures migrated from paper to electronic formats. It was therefore not possible to determine whether ePRO design best practices (e.g. those reported by the ePRO Consortium [17]) were applied in each implementation. In the future, we recommend that journal editors request screenshots illustrating the format of the migrated measure in associated supplementary online materials when accepting measurement comparability studies for publication. Despite these omissions, the results of these studies show broadly acceptable measurement comparability which is likely to be greater if the variability in implementation standards is eliminated through consistent application of best practices.

Many studies reported other forms of correlation as opposed to the ICC. In demonstrating comparability, it is more important to demonstrate a *y* = *x* relationship (as achieved by the ICC analysis) as opposed to a more general measure of correlation. The studies reporting ICC did not identify the type of ICC calculated, and this is important to be able to assess the adequacy of the reported analysis. In addition, test–retest reliability measures of the paper versions were not reported, making the determination of study-specific equivalence thresholds more difficult.

### Recommendations for Future VAS Use

The use of a VAS should be carefully evaluated for implementation in a clinical trial as some respondents may have difficulty understanding the scale or may be unable to use it because of visual or physical impairment.

The ePRO Consortium does not recommend the use of VASs for newly developed PROMs because of the aforementioned difficulties for some respondents and the inability to implement a VAS scale on a voice-based system (e.g. Interactive Voice Response System [IVRS]). While IVRS is less frequently used, in the future we may see greater adoption of voice assistants such as Amazon Alexa or Google Assistant for data collection in clinical studies.

When implementing the VAS electronically, there is sufficient evidence already published to support measurement comparability to a paper original measure if ePRO design best practices are followed.

## Conclusions

While a formal meta-analysis was not possible, this review is able to help answer the question of comparability between electronic and paper formats and the relevance of VAS length. Authors of 23 of the 24 studies evaluated concluded that pVAS and eVAS implementations were equivalent, and strong correlations and ICCs (> 0.7) were reported for 85.4% and 94.6% of individual scale comparisons, respectively. These strong comparability findings are despite the variety of technologies studied and the inability to assess differences between implementations against the now established best practices.

While eVAS lengths studied were as short as 21 mm on a feature phone [37] and 24 mm on a smartwatch [35], it is recommended that researchers should carefully consider the ability of users to accurately select marker positions on such small scales. For example, the smartwatch evaluation reported relatively low correlations (0.65 to 0.75) [35] compared to other studies. It is also recommended that care is taken to avoid scale-end effects (as seen with early devices such as Apple Newton and Palm Pilot) by ensuring easy access to and selection of responses at the scale ends. While modern smartphones and tablets typically have completely flat screens, care may be needed when the devices are provided with protective shells and covers.

We conclude, therefore, that there is sufficient evidence in the literature supporting the comparability of eVAS and pVAS regardless of the VAS length, participant age, or disease population. When implementing a VAS on a screen-based electronic mode, we recommend following industry best practices for faithful migration to minimise the likelihood of non-comparability to the pVAS.

## Abbreviations

BASDAI: Bath Ankylosing Spondylitis Disease Activity Index (Table 1)
eVAS: Visual analogue scale presented electronically
FDA: US Food and Drug Administration
FP: Feature phone (Table 1)
HAQ-DI: Health Assessment Questionnaire Disability Index (Table 1)
LCSS: Lung Cancer Symptom Scale (Table 1)
NRS: Numeric rating scale
PC: Personal computer (Table 1)
PDA: Personal digital assistant (Table 1)
PROM: Patient-reported outcome measure
pVAS: Visual analogue scale presented on paper
SF-MPQ: Short form McGill Pain Questionnaire (Table 1)
SP: Smartphone (Table 1)
VAS: Visual analogue scale
WOMAC: Western Ontario and McMaster Universities Osteoarthritis Index
